# A structured clinical nutrition pathway for enteral nutrition management in patients with intracerebral hemorrhage: a non-randomized controlled study

**DOI:** 10.3389/fnut.2026.1843128

**Published:** 2026-06-09

**Authors:** Nian Liu, Yanjiao Zheng, Jing Wen

**Affiliations:** Jiangjin Center Hospital of Chongqing, Chongqing, China

**Keywords:** clinical nutrition pathway, enteral nutrition, intracerebral hemorrhage, nutritional care, SAPIM

## Abstract

**Objective:**

To examine whether a SAPIM-based structured clinical nutrition pathway, operationalized in this study as systematic assessment, personalized intervention, and multidisciplinary management, was associated with improved enteral nutrition management and short-term clinical outcomes in patients with intracerebral hemorrhage (ICH).

**Methods:**

This non-randomized controlled study was conducted in a tertiary hospital in China between January and June 2025. A total of 60 patients with hypertensive intracerebral hemorrhage who required enteral nutrition were included. Patients in the intervention group received care under the SAPIM-based clinical nutrition pathway, while those in the control group received routine nutritional care. The primary outcome was the occurrence of enteral nutrition–related complications during hospitalization. Secondary outcomes included nutritional indicators (NRS-2002, prealbumin, albumin, total protein, and hemoglobin), functional outcomes (ADL, NIHSS, and aspiration risk score), patient satisfaction, and length of hospital stay.

**Results:**

Compared with the routine care group, the SAPIM group had a lower rate of enteral nutrition–related complications (30.0% vs. 56.7%, *p* = 0.037). Patients managed under the SAPIM pathway showed more favorable nutritional and functional trajectories during hospitalization, and their hospital stay was shorter than that of the control group (15.5 ± 4.9 vs. 23.4 ± 8.5 days, *p* < 0.0001). In exploratory logistic regression analyses, SAPIM implementation was associated with a lower risk of enteral nutrition–related complications in the unadjusted model and remained consistent in the primary adjusted and BMI-adjusted sensitivity models.

**Conclusion:**

The SAPIM-based clinical nutrition pathway was associated with safer and more consistent enteral nutrition management and more favorable short-term clinical profiles in patients with intracerebral hemorrhage. This structured approach may be a practical option for improving nutritional care in neurocritical settings.

## 1 Introduction

Intracerebral hemorrhage (ICH) is one of the most severe forms of stroke. Although it accounts for only about 10–15% of all stroke cases, it is associated with substantial early mortality, with nearly 30–50% of patients dying within the first month after onset (1). Among survivors, persistent neurological deficits are common, and many patients present with dysphagia, impaired consciousness, and metabolic disturbances, all of which may increase the risk of infection, complications, and poor functional recovery (2). Nutritional problems are therefore common in this population. Previous studies have shown that malnutrition frequently develops in the early stage after stroke, and inadequate nutritional intake may worsen catabolic stress and systemic inflammation, delay rehabilitation, and prolong hospitalization (1, 2). For these reasons, timely and sustained nutritional support has become an important part of comprehensive neurocritical care for patients with ICH.

Current clinical guidelines also highlight the importance of early nutritional management in stroke care. Recommendations from the European Society for Clinical Nutrition and Metabolism (ESPEN) and the American Society for Parenteral and Enteral Nutrition (ASPEN) emphasize early nutritional risk screening, individualized nutritional planning, and continued monitoring during treatment (3, 4). However, translating these recommendations into routine practice is often difficult. In many clinical settings, nutritional assessment is delayed, feeding practices are not fully standardized, and coordination among healthcare professionals remains inconsistent. Monitoring of enteral nutrition tolerance may also vary across wards and staff. These gaps in implementation can increase the risk of feeding-related complications and may compromise recovery in patients with ICH. Although previous studies suggest that structured enteral nutrition protocols may help improve metabolic stability and support neurological recovery after stroke, pathway-based nutritional management remains underused in real-world practice (5, 6).

In the present study, SAPIM was used as an operational acronym for a structured clinical nutrition pathway characterized by systematic assessment, personalized intervention, and multidisciplinary management. The pathway was developed with reference to standardized post-stroke nutrition management principles, clinical nutrition guidelines, and adult enteral nutrition nursing standards, and was adapted to the local neurocritical care workflow.

Although existing clinical nutrition guidelines emphasize early nutritional screening, individualized nutritional support, regular monitoring, and multidisciplinary collaboration, they often provide broad principles rather than detailed bedside workflows specifically tailored to patients with intracerebral hemorrhage requiring enteral nutrition (7). In routine practice, this may lead to variation in assessment timing, feeding adjustment, tube management, complication monitoring, and multidisciplinary communication. Therefore, the SAPIM-based pathway in the present study was designed to operationalize these guideline-based principles into a structured, nurse-led clinical nutrition process for patients with intracerebral hemorrhage. Whether a SAPIM-based clinical nutrition pathway can reduce enteral nutrition–related complications and improve short-term outcomes in this high-risk population remains unclear.

To address this gap, we developed and implemented the SAPIM-based structured clinical nutrition pathway as part of a hospital-based practice improvement initiative. The pathway was designed to improve the safety, continuity, and consistency of enteral nutrition management in patients with ICH through earlier risk identification, individualized nutritional support, and closer interdisciplinary collaboration. Related work has increasingly highlighted the value of standardized nutritional pathways, care bundles, and implementation-oriented strategies in stroke care and enteral nutrition practice (8–10). Against this background, the present study examined whether implementation of the SAPIM-based clinical nutrition pathway could reduce enteral nutrition–related complications and improve nutritional status and short-term clinical outcomes in patients with intracerebral hemorrhage, in line with established clinical nutrition guidance (11, 12).

## 2 Materials and methods

### 2.1 Study design and ethical considerations

This was a single-center, prospective, non-randomized controlled study conducted within a practice improvement framework. Eligible patients with hypertensive intracerebral hemorrhage requiring enteral nutrition were consecutively screened during the study period. To maintain intervention integrity and minimize cross-contamination between different care standards, group allocation was determined at the nursing unit level rather than through individual randomization.

During the study period, the SAPIM-based clinical nutrition pathway was implemented in a designated neurosurgical nursing unit after the responsible nursing staff had completed structured SAPIM-specific training and integrated the pathway into routine enteral nutrition care. Patients admitted to this unit were managed according to the SAPIM-based protocol and were included in the intervention group. In parallel, patients admitted to a comparable neurosurgical nursing unit, where the nursing staff continued to follow routine enteral nutrition care protocols and had not yet implemented the SAPIM pathway during the study period, were included in the control group. Admission to either unit was determined by institutional bed availability and standard clinical triage. The same eligibility criteria were applied to patients in both units. No individual-level random allocation, allocation concealment procedure, or blinding was used.

Ethical approval was obtained from the Ethics Committee of Jiangjin Center Hospital of Chongqing (approval no. KY2023055-A; approval date: October 11, 2024). Written informed consent was obtained from all participants before data collection. For patients who were temporarily unable to provide consent because of impaired consciousness or neurological deficits, consent was obtained from their legally authorized representatives in accordance with institutional policy and the Declaration of Helsinki (2013 revision) (13).

Clinical trial registration was not performed because the study was designed and approved as a single-center practice improvement study evaluating a structured process-of-care pathway implemented within routine clinical practice, rather than as a prospectively registered randomized clinical trial. All data were anonymized before analysis, and patient confidentiality was protected throughout the study.

### 2.2 Participants

Patients were eligible for inclusion if they met all of the following criteria: diagnosis of hypertensive intracerebral hemorrhage confirmed by cranial CT or MRI, with a hematoma volume of less than 30 mL; age between 18 and 80 years; Glasgow Coma Scale (GCS) score between 8 and 14 on admission; Nutritional Risk Screening 2002 (NRS-2002) score of 3 or higher; dysphagia requiring enteral nutrition through a nasogastric or nasojejunal tube; and an expected hospital stay of at least 48 h. The nutritional risk threshold was based on established clinical nutrition assessment practice (14).

Patients were excluded if they had pre-existing enteral nutrition–related complications, such as diarrhea, gastric retention, or aspiration; severe cardiac, hepatic, or renal failure; hemodynamic instability; or malignant disease. Patients who were transferred to other departments during hospitalization or had incomplete core outcome data required for the primary analysis were also excluded from the final analysis.

A total of 60 patients met the eligibility criteria and were included in the study, with 30 patients in the control group and 30 in the intervention group. Eligible patients admitted during the study period were consecutively enrolled and included if they met the predefined criteria and had complete outcome data. Additional baseline variables, including body mass index (BMI), admission C-reactive protein (CRP), and selected major comorbidities and baseline clinical conditions, were extracted from the electronic medical records when available. BMI was calculated from recorded admission height and weight. Admission CRP was defined as the earliest available CR *p* value during early hospitalization. Variables with incomplete availability were summarized using available cases, and missing values were not imputed.

### 2.3 Intervention

At enrollment, all included patients had dysphagia or impaired consciousness requiring enteral nutrition through a nasogastric or nasojejunal tube. Enteral nutrition was initiated within 48 h after admission and was the primary planned route of nutritional support during the study observation period. Oral intake, if resumed after clinical swallowing reassessment during hospitalization, was determined by the treating team according to swallowing safety and clinical status. However, actual oral intake volume and total nutritional intake were not systematically quantified in the analytic dataset.

#### 2.3.1 Control group (routine care)

Patients in the control group received standard enteral nutrition care. This included verification of the feeding formula, confirmation of tube placement and insertion length, administration of enteral feeding via controlled gravity infusion at 15–20 drops/min or via a feeding pump according to clinical condition and feeding tolerance, routine tube maintenance, and basic education for patients and caregivers. Enteral nutrition was typically initiated within 48 h after hospital admission.

#### 2.3.2 Intervention group (SAPIM pathway)

Patients in the intervention group were managed using a SAPIM-based clinical nutrition pathway that was operationalized in this study as a structured process involving systematic assessment, personalized intervention, multidisciplinary management, tube management and early warning, and monitoring with feedback.

**Figure 1:**
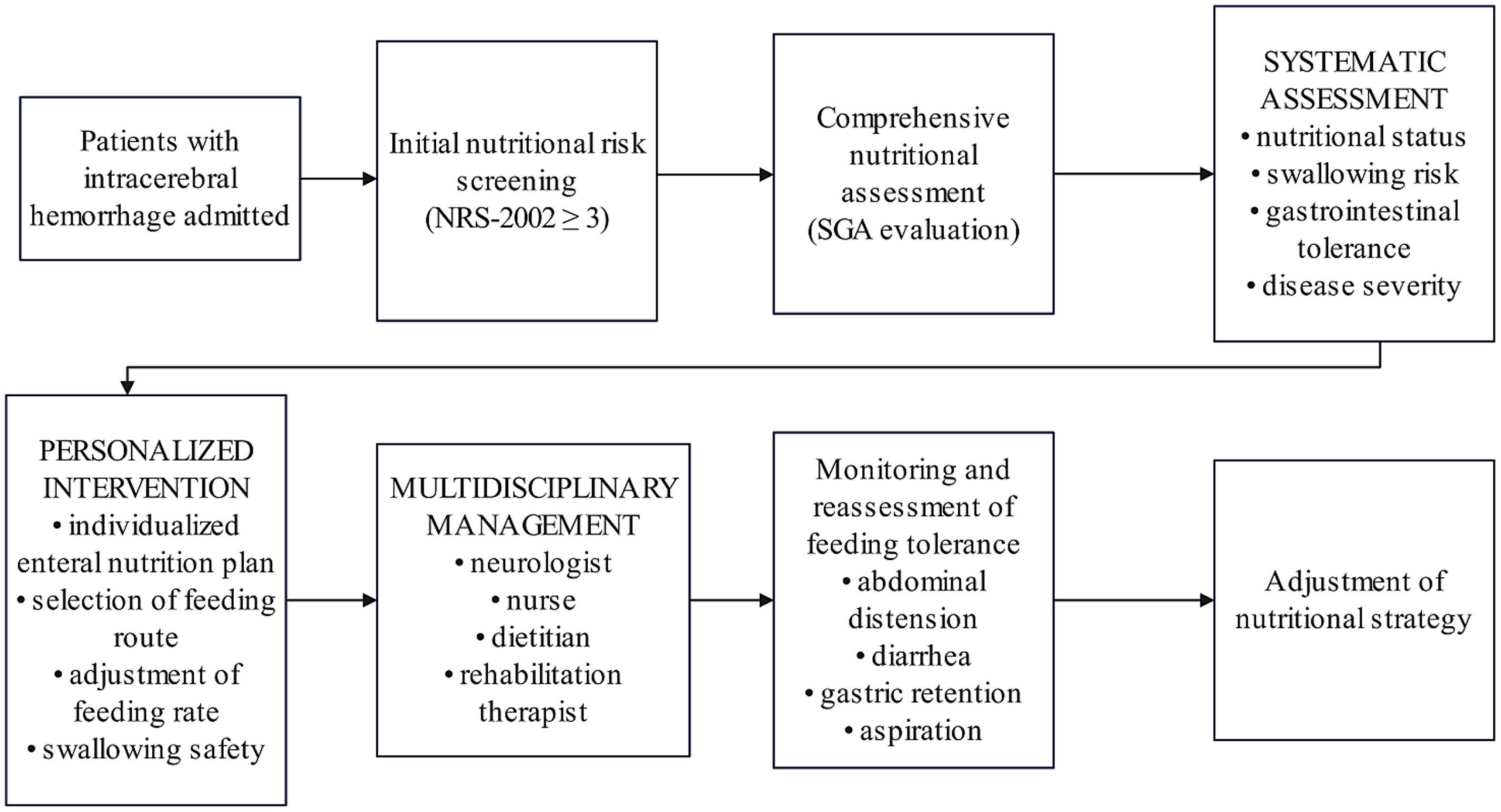
Clinical workflow of the SAPIM nutritional management pathway for patients with intracerebral hemorrhage.

The overall workflow of the SAPIM pathway is shown in Figure 1.

The pathway consisted of five key components, as defined in the present study:

#### 2.3.3 Systematic assessment

Within 24 h of admission, patients underwent a comprehensive clinical and nutritional assessment. This included nutritional risk screening using the Nutritional Risk Screening 2002 (NRS-2002), evaluation of nutritional status using the Subjective Global Assessment (SGA), assessment of aspiration risk, measurement of biochemical indicators (prealbumin, albumin, total protein, and hemoglobin), and functional evaluation using NIHSS and ADL scales (15, 16). Nurses responsible for SGA assessment received standardized training, and a clinical dietitian provided supervision during implementation. The Global Leadership Initiative on Malnutrition (GLIM) criteria were not applied, as the pathway followed the hospital’s existing clinical nutrition assessment protocol.

#### 2.3.4 Personalized intervention

Individualized targets for energy, protein, and fluid intake were determined for each patient. Estimated energy requirements were calculated according to body weight and clinical condition, with a general target of approximately 25–30 kcal/kg/day during the first week of enteral nutrition. Protein targets were generally set at approximately 1.0–1.5 g/kg/day, depending on disease severity, gastrointestinal tolerance, renal function, and the judgment of the treating physicians and dietitians. Feeding was advanced gradually during the early phase, with adjustment according to feeding tolerance, gastric retention, abdominal distension, diarrhea, aspiration risk, and biochemical indicators. The SAPIM pathway emphasized individualized nutritional goal setting, close monitoring, and timely adjustment rather than a fixed formula-based intervention. Enteral nutrition formulas were prescribed according to routine clinical practice based on each patient’s nutritional needs, gastrointestinal tolerance, and the judgment of the treating physicians and dietitians. The SAPIM pathway itself did not require, standardize, or compare any specific formula containing n-3 polyunsaturated fatty acids, oligosaccharides, probiotics, or other specialized immunonutrients. Enteral feeding was delivered according to a standardized protocol, including controlled formula temperature (37 ± 1 °C), caloric density (≤1.5 kcal/mL), and controlled infusion by gravity infusion or feeding pump according to feeding tolerance. Therefore, the intervention should be interpreted as a structured nutritional care pathway rather than a formula-specific nutritional intervention.

#### 2.3.5 Multidisciplinary management

Nurses worked in coordination with physicians, dietitians, and rehabilitation therapists to optimize feeding tolerance, prevent aspiration, and manage patient positioning. Additional nutrition-related treatments, such as albumin supplementation, amino acid infusion, or parenteral nutrition, were prescribed by treating physicians based on clinical indications. No study-specific pharmacological nutritional interventions were introduced beyond the SAPIM pathway.

#### 2.3.6 Tube management and early warning

Tube fixation and skin condition were checked daily. A two-person verification process was used to ensure correct tube placement. Early warning procedures were applied to identify patients at higher risk of tube-related complications.

#### 2.3.7 Monitoring and feedback

Patients were reassessed every three days, and the nutritional plan was adjusted according to clinical progress. Regular team discussions and case reviews were conducted to support ongoing pathway implementation.

Before discharge, individualized education and follow-up guidance were provided to patients and caregivers to support continuity of nutritional care.

### 2.4 Outcomes

#### 2.4.1 Primary outcome

The primary outcome was the occurrence of any enteral nutrition–related complication during hospitalization. If a patient experienced more than one type of complication, the patient was counted once when calculating the overall incidence, while each specific complication was recorded separately for descriptive analysis. Diagnostic criteria for enteral nutrition–related complications were based on the Clinical Nutrition Nursing Standards of the Chinese Society for Parenteral and Enteral Nutrition (2021 edition).

#### 2.4.2 Secondary outcomes

Secondary outcomes included the following:

(1) Nutritional indicators: NRS-2002 score, prealbumin, albumin, total protein, and hemoglobin, assessed at admission, day 3, day 7, and discharge.
(2) Functional and safety outcomes included Activities of Daily Living (ADL), National Institutes of Health Stroke Scale (NIHSS), and aspiration risk score assessed using the hospital-based Neurosurgical Patient Aspiration Risk Assessment Form. This form includes eight assessment domains: age, level of consciousness, sputum status, relevant neurological comorbidities, diet, body position, water-swallowing test, and artificial airway or mechanical ventilation. Based on the item scoring, the theoretical total score range is 8–23. According to the institutional risk classification, scores of 10–12, 13–18, and 19–23 indicate low, moderate, and high aspiration risk, respectively. Higher scores indicate greater aspiration risk.
(3) Unplanned tube removal: defined as self-extubation or accidental tube dislodgement during hospitalization.
(4) Patient satisfaction: assessed at discharge using a validated nursing satisfaction questionnaire (Cronbach’s *α* = 0.85).
(5) Length of hospital stay: total duration of hospitalization, calculated from admission to discharge.

### 2.5 Statistical analysis

All statistical analyses were performed using SPSS software (version 26.0; IBM Corp., Armonk, NY, United States). Continuous variables are presented as mean ± standard deviation (SD) or median and interquartile range (IQR), as appropriate. For repeated continuous outcomes measured at admission, day 3, day 7, and discharge, linear mixed-effects models were used to evaluate the effects of group, time, and the group × time interaction while accounting for within-patient correlations across repeated measurements. Patient ID was included as a random effect, and group, time, and group × time interaction were included as fixed effects. Time was treated as a categorical variable. The group × time interaction was used to assess whether changes over time differed between the control and intervention groups. Between-group comparisons at individual time points were retained as descriptive *post hoc* comparisons to aid clinical interpretation. Categorical variables are presented as frequencies and percentages and were analyzed using the chi-square test or Fisher’s exact test, as appropriate. CRP was summarized as median (IQR) because of its skewed distribution and was compared between groups using the Mann–Whitney U test. Because albumin differed between groups at admission, an additional sensitivity analysis was performed for post-baseline albumin values using a baseline-adjusted linear mixed-effects model with admission albumin included as a covariate.

Given the limited sample size, exploratory unadjusted and adjusted logistic regression analyses were performed to examine the association between SAPIM pathway implementation and enteral nutrition–related complications. The unadjusted model included SAPIM pathway implementation only. The primary adjusted model included SAPIM pathway implementation, age, sex, and Glasgow Coma Scale (GCS) score as clinically relevant covariates. Because BMI was available for all participants and is clinically relevant to nutritional status, an additional BMI-adjusted sensitivity logistic regression model was performed by adding BMI to the primary adjusted model. Admission CRP and selected comorbidities and baseline clinical conditions were reported in the baseline characteristics table when available but were not incorporated into the primary adjusted logistic regression model because of incomplete availability, sparse distributions, the limited sample size, and the small number of outcome events. To avoid model instability, the primary adjusted model was kept parsimonious. Odds ratios (ORs), adjusted odds ratios (aORs), and 95% confidence intervals (CIs) were reported. Because of the small sample size, adjusted estimates were interpreted cautiously and were considered exploratory.

All statistical tests were two-tailed, and a *p* value < 0.05 was considered statistically significant.

## 3 Results

### 3.1 Baseline characteristics

A total of 60 patients with intracerebral hemorrhage (ICH) were included in the study, with 30 patients in the control group and 30 in the intervention group. All eligible patients admitted during the study period were included in the final analysis.

After additional review of the electronic medical records, BMI, admission CRP, and selected major comorbidities and baseline clinical conditions were added to the baseline characteristics table when available. BMI data were available for all 60 participants, whereas CRP data were available for 40 of 60 participants. No statistically significant between-group differences were observed in sex, age, hematoma volume, GCS score, NRS-2002 eligibility status, level of consciousness, feeding tube type, NIHSS score at admission, BMI, available admission CRP, or the selected comorbidities and baseline clinical conditions summarized in Table 1. However, given the small sample size, incomplete availability of CRP, and non-randomized nursing unit-level design, the absence of statistical significance should not be interpreted as evidence of complete baseline equivalence.

**Table 1:** Comparison of baseline characteristics between the control group (*n* = 30) and the intervention group (*n* = 30).

Variable	Control group (*n* = 30)	Intervention group (*n* = 30)	Statistic	*p* value
Sex (male/female)	18/12	20/10	χ^2^ = 0.278	0.598
Age (years)	58.87 ± 13.56	64.43 ± 9.59	t = 1.836	0.075
Hematoma volume (mL)	18.77 ± 5.34	20.57 ± 4.75	t = 1.390	0.170
GCS score	10.27 ± 2.39	10.10 ± 2.20	t = 0.279	0.781
NRS-2002 score	≥3 (all)	≥3 (all)	—	—
Level of consciousness, somnolence/stupor/light coma	4/11/15	2/13/15	χ^2^ = 0.833	0.659
Feeding tube type, nasogastric/nasojejunal	29/1	25/5	χ^2^ = 2.963	0.085
NIHSS score at admission	16.07 ± 5.29	15.40 ± 4.57	t = 0.523	0.603
BMI, kg/m^2^	23.56 ± 3.42	22.93 ± 3.19	t = 0.745	0.459
Admission CRP, mg/L, median (IQR)	70.28 (9.30–107.69), *n* = 18	46.92 (21.87–110.06), *n* = 22	U = 174.0	0.881
Documented history of hypertension, *n* (%)	21 (70.0)	23 (76.7)	—	0.771
Diabetes mellitus, *n* (%)	2 (6.7)	4 (13.3)	—	0.671
Pulmonary infection or pneumonia at admission, *n* (%)	21 (70.0)	26 (86.7)	—	0.209
Coronary heart disease or coronary stent, *n* (%)	0 (0.0)	2 (6.7)	—	0.492
Chronic pulmonary disease, *n* (%)	2 (6.7)	2 (6.7)	—	1.000
Kidney disease, *n* (%)	0 (0.0)	3 (10.0)	—	0.237
Prior cerebral infarction/stroke-related diagnosis, *n* (%)	1 (3.3)	1 (3.3)	—	1.000

BMI was available for all participants. CRP data were available for 40 of 60 participants and were summarized using available cases. Values recorded as >200 mg/L or <0.5 mg/L were treated as boundary values for descriptive analysis. Missing CR *p* values were not imputed. CRP was compared using the Mann–Whitney U test. Selected comorbidities and baseline clinical conditions were extracted from electronic medical records and compared using Fisher’s exact test where appropriate.

All participants met the diagnostic criteria for hypertensive intracerebral hemorrhage; the hypertension row refers to a documented prior history of hypertension in the electronic medical records.

### 3.2 Enteral nutrition–related complications

The incidence of enteral nutrition–related complications was lower in the SAPIM group than in the control group (9/30 [30.0%] vs. 17/30 [56.7%]; *p* = 0.037).

To further explore the association between SAPIM pathway implementation and enteral nutrition–related complications, exploratory unadjusted and adjusted logistic regression analyses were performed. In the unadjusted logistic regression analysis, SAPIM pathway implementation was associated with a lower risk of enteral nutrition–related complications (OR = 0.33, 95% CI: 0.11–0.95, *p* = 0.040). The direction of the association remained consistent after adjustment for age, sex, and GCS score (aOR = 0.40, 95% CI: 0.15–0.98, *p* = 0.045). In an additional BMI-adjusted sensitivity model, the association between SAPIM pathway implementation and enteral nutrition–related complications remained directionally consistent after further adjustment for BMI (aOR = 0.42, 95% CI: 0.17–0.99, *p* = 0.048) (Table 2). Given the limited sample size, all adjusted estimates should be interpreted cautiously and considered exploratory.

**Table 2:** Unadjusted and adjusted associations between SAPIM pathway implementation and enteral nutrition–related complications.

Model name	Variable	OR/aOR	95% CI	*p* value
Unadjusted model	SAPIM pathway implementation vs. routine care	0.33	0.11–0.95	0.040
Primary adjusted model	SAPIM pathway implementation vs. routine care	0.40	0.15–0.98	0.045
BMI-adjusted sensitivity model	SAPIM pathway implementation vs. routine care	0.42	0.17–0.99	0.048

The primary adjusted model included age, sex, and GCS score as covariates. The BMI-adjusted sensitivity model additionally included BMI. OR, odds ratio; aOR, adjusted odds ratio; CI, confidence interval; GCS, Glasgow Coma Scale. Given the limited sample size, adjusted estimates were interpreted cautiously and were considered exploratory.

### 3.3 Unplanned tube removal

Unplanned tube removal, defined as self-extubation or accidental tube dislodgement, occurred in 3 patients in the SAPIM group and 8 patients in the control group (10.0% vs. 26.7%). Therefore, although unplanned tube removal was numerically less frequent in the SAPIM group, the difference did not reach statistical significance.

### 3.4 Patient satisfaction

Patient satisfaction responses are descriptively summarized as follows: in the SAPIM group, 29 patients were very satisfied and 1 was generally satisfied, whereas in the control group, 21 were very satisfied, 5 were generally satisfied, and 4 were dissatisfied.

### 3.5 Nutritional status indicators

Nutritional indicators changed over time in both groups, with greater improvement observed in the SAPIM group. Linear mixed-effects models showed significant group × time interaction effects for NRS-2002 score, prealbumin, albumin, total protein, and hemoglobin (all P for interaction < 0.001), indicating that the trajectories of nutritional indicators differed between the control and intervention groups over time. Descriptive between-group comparisons at individual time points are presented in Table 3 to aid clinical interpretation. A descriptive between-group difference in albumin was observed at admission; therefore, subsequent albumin trajectories should be interpreted with caution. In an additional baseline-adjusted linear mixed-effects model for post-baseline albumin values, the intervention group remained associated with higher post-baseline albumin levels after adjustment for admission albumin (*β* = 12.88 g/L, 95% CI: 10.63–15.12, *p* < 0.001).

**Table 3:** Comparison of NRS-2002 scores and laboratory parameters between control and intervention groups over time.

Variable	Time point	Control group (mean ± SD)	Intervention group (MEAN ± SD)	t	*p* value	P for group × time interaction
NRS-2002 score	At admission	3.50 ± 0.90	3.73 ± 0.94	0.9795	0.3314	<0.001
	Day 3	3.40 ± 0.68	2.40 ± 0.77	5.3500	<0.0001	
	Day 7	3.33 ± 0.80	2.17 ± 0.70	6.0050	<0.0001	
	At discharge	3.17 ± 0.70	2.13 ± 0.68	5.7980	<0.0001	
Prealbumin (mg/L)	At admission	306.20 ± 62.33	328.90 ± 77.88	1.2460	0.2176	<0.001
	Day 3	209.20 ± 51.96	356.00 ± 65.89	9.5818	<0.0001	
	Day 7	231.70 ± 52.39	356.30 ± 48.95	9.5160	<0.0001	
	At discharge	247.33 ± 55.16	371.32 ± 39.85	9.9810	<0.0001	
Albumin (g/L)	At admission	43.08 ± 4.39	45.33 ± 3.81	2.1229	0.038	<0.001
	Day 3	31.77 ± 6.85	45.60 ± 4.32	9.4140	<0.0001	
	Day 7	31.75 ± 6.60	46.07 ± 3.62	10.4100	<0.0001	
	At discharge	33.13 ± 5.27	46.25 ± 3.15	11.7000	<0.0001	
Total protein (g/L)	At admission	72.33 ± 5.12	70.90 ± 7.14	0.8970	0.3749	<0.001
	Day 3	62.67 ± 4.35	68.80 ± 5.58	4.7930	<0.0001	
	Day 7	65.22 ± 5.56	70.06 ± 4.71	3.6430	0.0006	
	At discharge	63.90 ± 6.34	71.96 ± 3.75	5.999	<0.0001	
Hemoglobin (g/L)	At admission	132.60 ± 19.60	139.50 ± 14.81	1.5460	0.1276	<0.001
	Day 3	116.70 ± 16.87	137.60 ± 8.77	6.0210	<0.0001	
	Day 7	111.50 ± 15.46	134.20 ± 5.34	7.6020	<0.0001	
	At discharge	110.90 ± 11.36	130.00 ± 5.95	8.1720	<0.0001	

Values are presented as mean ± SD. Descriptive *p* values at individual time points are shown for clinical interpretation. The longitudinal group × time interaction was tested using a linear mixed-effects model. Because albumin differed between groups at admission, an additional baseline-adjusted sensitivity analysis was performed for post-baseline albumin values.

### 3.6 Functional and safety outcomes

Functional and safety outcomes changed over time in both groups, with more favorable trajectories observed in the SAPIM group. Linear mixed-effects models showed significant group × time interaction effects for aspiration risk score, ADL score, and NIHSS score (all P for interaction < 0.001), indicating that changes in aspiration risk, daily living ability, and neurological impairment differed between the control and intervention groups over time. Descriptive between-group comparisons at individual time points are presented in Table 4 to aid clinical interpretation.

**Table 4:** Comparison of aspiration risk, ADL, and NIHSS scores between the control and intervention groups over time.

Variable	Time point	Control group (mean ± SD)	Intervention group (mean ± SD)	t	*p* value	P for group × time interaction
Aspiration risk score	At admission	16.90 ± 3.51	16.00 ± 3.87	0.9441	0.3491	<0.001
	Day 3	16.23 ± 3.36	13.63 ± 3.68	2.8570	0.0059	
	Day 7	14.23 ± 2.86	11.33 ± 2.22	4.3880	<0.0001	
	At discharge	13.67 ± 2.34	10.30 ± 0.75	7.5070	<0.0001	
ADL score	At admission	8.67 ± 9.19	7.50 ± 7.40	0.5418	0.5900	<0.001
	Day 3	9.50 ± 9.77	19.17 ± 8.52	4.0850	0.0001	
	Day 7	12.00 ± 9.34	37.17 ± 13.04	8.5920	<0.0001	
	At discharge	18.50 ± 11.15	43.17 ± 15.56	7.056	<0.0001	
NIHSS score	At admission	16.07 ± 5.29	15.40 ± 4.57	0.5227	0.6032	<0.001
	Day 3	15.80 ± 5.14	12.63 ± 4.12	2.6320	0.0109	
	Day 7	14.63 ± 4.81	8.07 ± 3.70	5.9300	<0.0001	
	At discharge	12.53 ± 4.35	3.63 ± 2.17	10.0200	<0.0001	

Values are presented as mean ± SD. Descriptive *p* values at individual time points are shown for clinical interpretation. The longitudinal group × time interaction was tested using a linear mixed-effects model.

### 3.7 Length of hospital stay

The mean length of hospital stay was significantly shorter in the SAPIM group than in the control group (15.50 ± 4.92 days vs. 23.40 ± 8.50 days; t = 4.408, *p* < 0.0001) (Table 5).

**Table 5:** Comparison of length of hospital stay between control and intervention groups.

Group	*n*	Length of hospital stay (days, mean ± SD)	t	*p*
Control group	30	23.40 ± 8.50	4.408	<0.0001
Intervention group	30	15.50 ± 4.92		

## 4 Discussion

In this hospital-based non-randomized study, implementation of the SAPIM-based clinical nutrition pathway was associated with safer and more consistent enteral nutrition management in patients with intracerebral hemorrhage (ICH). Compared with routine care, the SAPIM group had a lower rate of enteral nutrition–related complications, together with more favorable nutritional and functional outcomes and a shorter length of hospital stay. These findings suggest that a structured clinical nutrition pathway incorporating systematic assessment, individualized intervention, and multidisciplinary collaboration may help improve the quality and consistency of nutritional care in this high-risk population.

### 4.1 Mechanisms for reducing enteral nutrition–related complications

The lower rate of enteral nutrition–related complications observed in the SAPIM group may be related to several features of the pathway. First, the SAPIM-based pathway emphasizes early risk identification, targeted intervention, and regular reassessment. This structured process may have helped the clinical team detect potential problems earlier and respond more consistently during enteral nutrition management.

A second likely explanation is the standardization of key bedside practices, including tube placement verification, fixation, and routine maintenance. These measures are important for reducing mechanical problems and procedure-related errors and are consistent with current recommendations for nasogastric tube management in adult patients (17). In addition, maintaining a semi-recumbent position with appropriate head-of-bed elevation is widely recognized as an important strategy for reducing aspiration risk. Previous studies have shown that supine positioning is associated with a higher risk of aspiration and pneumonia, whereas head elevation can reduce aspiration-related events (18, 19).

Another important feature of the SAPIM pathway is that these measures were implemented as part of an integrated care process rather than as isolated actions. In routine practice, enteral nutrition complications are often managed only after they become clinically apparent. In contrast, the SAPIM pathway supports a more proactive approach by combining risk assessment, feeding management, tube care, and ongoing monitoring within the same pathway. This may have contributed to better feeding safety and greater stability during hospitalization.

It is also possible that the reduction in feeding-related complications was linked to more consistent nutritional management processes. Early nutritional screening and individualized feeding plans may have supported more consistent nutritional decision-making during the acute stage of illness, although actual energy and protein delivery were not systematically quantified in this study. More structured nutritional support may in turn have created more favorable conditions for feeding safety, rehabilitation participation, and overall clinical stabilization. However, these potential mechanisms should be interpreted cautiously, as the present study was not designed to determine causality.

### 4.2 Clinical implementation of nutritional optimization

The observed changes in NRS-2002 scores and biochemical indicators, including prealbumin, albumin, total protein, and hemoglobin, suggest that the SAPIM pathway may have been associated with more favorable short-term nutritional profiles during hospitalization. In patients with intracerebral hemorrhage, adequate delivery of energy and protein is important for maintaining metabolic stability and limiting the nutritional consequences of acute illness. Early use of NRS-2002 may also help identify patients at nutritional risk and support the timely initiation of individualized nutritional support.

Within the SAPIM-based pathway, nutritional management was implemented through a structured multidisciplinary process. Standardized protocols were used to guide estimation of nutritional requirements, adjustment of feeding regimens, and monitoring of feeding tolerance, while communication among clinicians, nurses, dietitians, and rehabilitation staff was maintained throughout hospitalization. This approach is consistent with previous evidence suggesting that protocolized enteral nutrition strategies, particularly those addressing feeding timing, infusion rate, and formula selection, may improve feeding tolerance and metabolic outcomes in critically ill and stroke populations (20–22).

Taken together, these findings suggest that structured nutritional protocols combined with multidisciplinary collaboration may help improve the consistency and practicality of nutritional care in routine neurocritical practice.

### 4.3 Functional recovery and safe rehabilitation

The more favorable aspiration-safety-related, neurological, and functional outcomes observed in the SAPIM group suggest that optimized nutritional management may help create more favorable conditions for rehabilitation after intracerebral hemorrhage. The use of structured clinical assessments, including the National Institutes of Health Stroke Scale (NIHSS), Activities of Daily Living (ADL) assessment, and aspiration risk assessment, allowed patient progress to be monitored consistently during hospitalization.

Structured aspiration risk assessment, together with aspiration-prevention measures such as head-of-bed elevation, feeding tolerance monitoring, tube-position verification, and timely nursing reassessment, may support safer enteral nutrition management and help reduce aspiration-related complications (23). The marked decrease in NIHSS scores observed in the SAPIM group should be interpreted cautiously. After rechecking the individual-level NIHSS data at admission, day 3, day 7, and discharge, the values reported in Table 4 were confirmed. No data-entry error, scoring reversal, or column misclassification was identified. However, the magnitude of NIHSS improvement in the SAPIM group remains unexpectedly large and cannot be fully explained by the variables available in this study. Unmeasured factors, such as hematoma location and evolution, perihematomal edema resolution, rehabilitation intensity, medical treatment differences, discharge timing, and potential inter-rater variability in NIHSS assessment, may have contributed to this finding. Therefore, the NIHSS result should be interpreted as an exploratory short-term functional observation rather than evidence of a direct neurological effect of the SAPIM pathway. When nutritional support is coordinated with aspiration-prevention strategies and rehabilitation training, patients may be better able to tolerate feeding safely and participate in recovery activities.

For this reason, the improvements observed in NIHSS scores, ADL performance, and aspiration-risk-related indicators should be interpreted as supportive associations rather than evidence of a direct causal relationship.

### 4.4 Reduced hospitalization and clinical implications

In this context, the shorter hospital stay in the SAPIM group may be related to improved feeding safety, closer monitoring, and earlier clinical stabilization. Previous studies have shown that malnutrition and delayed nutritional intervention are associated with prolonged hospitalization and higher healthcare use in patients with stroke (24). Nevertheless, discharge timing may also have been influenced by non-nutritional factors, including institutional workflow and clinical decision-making.

From a clinical perspective, these findings support the value of incorporating standardized nutritional pathways into routine care for patients with intracerebral hemorrhage. A pathway-based approach may help improve the consistency of enteral nutrition management and reduce variation in bedside practice. In settings where nutritional assessment and feeding management are not yet fully standardized, such an approach may offer a practical framework for improving care delivery.

### 4.5 Clinical significance and generalizability

The present study suggests that a structured, pathway-based approach to enteral nutrition management is feasible in routine neurocritical care for patients with intracerebral hemorrhage. In this study, the SAPIM pathway appeared to address several common challenges in conventional nutritional care, including inconsistent risk assessment, variation in feeding practice, and limited coordination across disciplines. By providing a more standardized approach to enteral nutrition management, the pathway may help improve the consistency of care during hospitalization.

The SAPIM-based pathway is relatively simple in structure and does not rely on highly specialized equipment or complex additional resources. For this reason, it may have potential applicability in other stroke units or clinical settings caring for high-risk neurological patients, provided that local staffing patterns, nursing workflows, and multidisciplinary resources are considered. However, its generalizability should be interpreted with caution, as the present study was conducted at a single center and within a specific clinical workflow. Further validation in larger and more diverse settings would be needed before broader conclusions can be drawn.

Compared with routine enteral nutrition care, the SAPIM-based pathway provided several operational features that may explain the observed improvements. Nutritional risk screening and reassessment were embedded into a predefined workflow, individualized nutritional goals and feeding adjustments were linked to gastrointestinal tolerance and aspiration risk, tube management and complication monitoring were integrated into daily nursing practice, and multidisciplinary communication was supported through regular reassessment and feedback. These features may have improved the consistency and timeliness of enteral nutrition care. However, the present study was not designed to directly compare SAPIM with other established nutritional management frameworks or guideline-based care bundles.

### 4.6 Limitations and future directions

Several limitations should be considered when interpreting the findings of this study. First, this was a single-center, non-randomized nursing unit-level controlled study rather than a randomized controlled trial. Although group allocation at the nursing unit level helped maintain intervention integrity and reduce cross-contamination, unit-level confounding could not be fully excluded. Differences in nursing experience, staffing patterns, ward workflow, or unmeasured patient characteristics may have influenced the observed outcomes. In addition, although no statistically significant baseline differences were observed in the baseline clinical characteristics presented in Table 1, the relatively small sample size limited the ability to detect clinically meaningful imbalance. Although baseline albumin was considered in a sensitivity analysis, the admission imbalance may still have influenced the interpretation of albumin-related findings. Therefore, albumin results should be interpreted as supportive changes in short-term nutritional profiles rather than definitive evidence of a treatment effect. For example, the difference in age between groups may still have influenced complication risk, recovery trajectory, and length of hospital stay. Therefore, residual confounding due to measured and unmeasured baseline characteristics could not be excluded. Because of the limited sample size and number of outcome events, the adjusted logistic regression analysis should be interpreted as exploratory rather than confirmatory.

Furthermore, the relatively small sample size and single-center setting may limit the generalizability of the findings. Outcome assessment was limited to the hospitalization period, and longer-term outcomes, such as readmission, survival, and quality of life, were not evaluated. After additional review of the electronic medical records, BMI, admission CRP, and selected major comorbidities and baseline clinical conditions were extracted when available and added to Table 1. BMI was available for all participants, whereas admission CRP was available for 40 of 60 participants and was summarized using available cases without imputation. However, smoking status and alcohol use were not consistently retrievable. Therefore, residual confounding related to incomplete inflammatory, lifestyle, and comorbidity data could not be excluded, and our ability to fully adjust for these factors remained limited. In addition, neurological outcomes were assessed only during hospitalization, and rehabilitation intensity, hematoma evolution, and other neurological treatment-related factors were not systematically quantified. The marked decrease in NIHSS scores observed in the SAPIM group could not be fully explained by the available clinical variables and may reflect unmeasured neurological, imaging, treatment-related, rehabilitation-related, or assessment-related factors. Although the values were confirmed against the individual-level dataset, this finding should be interpreted with particular caution as an exploratory short-term functional observation rather than evidence of a direct neurological effect of the nutritional pathway.

In addition, nutritional assessment in this study relied mainly on biochemical indicators and nutritional risk screening tools. Longitudinal body weight change, muscle mass-related parameters, and detailed systemic inflammatory response syndrome data were not consistently available. Therefore, although BMI and available admission CRP were added to the baseline characteristics table, we could not evaluate longitudinal anthropometric changes or fully assess the influence of systemic inflammatory status on nutritional indicators and clinical outcomes.

Actual oral intake, enteral intake, and total nutritional intake were not systematically quantified for all participants. Therefore, although nutritional targets were set according to body weight and clinical condition, the actual achievement of energy and protein targets could not be fully assessed, and the potential contribution of resumed oral intake to changes in nutritional indicators could not be determined.

The study also did not systematically compare different enteral nutrition formula compositions. Although formula selection followed routine clinical practice, future studies should record formula composition in greater detail and evaluate whether specific nutrients, such as n-3 polyunsaturated fatty acids, oligosaccharides, probiotics, or other specialized immunonutrients, influence nutritional and clinical outcomes.

In addition, aspiration risk was assessed using a hospital-based standardized nursing risk assessment protocol rather than an internationally validated swallowing function scale. Although this tool was routinely used in clinical nursing practice and was applied consistently across both groups, its use may limit comparability with studies using validated dysphagia scales. Future studies should incorporate more comprehensive nutritional assessment to better clarify the relationship between structured nutritional management and clinical recovery (25).

Another limitation is that adherence to the SAPIM pathway was not formally measured. Variation in implementation may therefore have influenced the observed outcomes. Future research should include larger multicenter studies, longer follow-up, and more detailed evaluation of pathway fidelity to confirm the robustness and broader applicability of this approach. Further refinement of enteral nutrition protocols may also help improve the effectiveness of structured nutritional management in patients with intracerebral hemorrhage.

## 5 Conclusion

Implementation of the SAPIM-based clinical nutrition pathway was associated with safer and more consistent enteral nutrition management in patients with intracerebral hemorrhage. Compared with routine care, the SAPIM pathway was associated with fewer feeding-related complications, more favorable nutritional and functional trajectories, and a shorter length of hospital stay.

These findings support the potential value of a structured clinical nutrition pathway for improving the safety and consistency of enteral nutrition care in neurocritical settings. Further multicenter studies with larger sample sizes and longer follow-up are needed to confirm these findings and to better define the longer-term clinical value of this approach.

## Data Availability

The raw data supporting the conclusions of this article will be made available by the authors, without undue reservation.
